# Early Exercise Protects against Cerebral Ischemic Injury through Inhibiting Neuron Apoptosis in Cortex in Rats

**DOI:** 10.3390/ijms14036074

**Published:** 2013-03-15

**Authors:** Pengyue Zhang, Yuling Zhang, Jie Zhang, Yi Wu, Jie Jia, Junfa Wu, Yongshan Hu

**Affiliations:** 1 Medical Faculty, Kunming University of Science and Technology, Kunming 650500, China; E-Mail: kmzhjie@yahoo.cn (J.Z.); 2 Department of Rehabilitation, Huashan Hospital, Fudan University, Shanghai 200040, China; E-Mails: zhangyuling1982@hotmail.com (Y.Z.); wuyi3000@yahoo.com.cn (Y.W.); shannonjj@126.com (J.J.); junfawu2002@yahoo.com.cn (J.W.); 3 Yunnan Provincial Key Laboratory for Birth Defects and Genetic Diseases, Genetic Diagnosis Center, the First People’s Hospital of Yunnan Province, Kunming 650032, China; 4 The Yonghe Branch of Huashan Hospital, Fudan University, Shanghai 200436, China

**Keywords:** early exercise, cerebral ischemia, apoptosis, neuroprotection, caspase

## Abstract

Early exercise is an effective strategy for stroke treatment, but the underlying mechanism remains poorly understood. Apoptosis plays a critical role after stroke. However, it is unclear whether early exercise inhibits apoptosis after stroke. The present study investigated the effect of early exercise on apoptosis induced by ischemia. Adult SD rats were subjected to transient focal cerebral ischemia by middle cerebral artery occlusion model (MCAO) and were randomly divided into early exercise group, non-exercise group and sham group. Early exercise group received forced treadmill training initiated at 24 h after operation. Fourteen days later, the cell apoptosis were detected by TdT-mediated dUTP-biotin nick-end labeling (TUNEL) and Fluoro-Jade-B staining (F-J-B). Caspase-3, cleaved caspase-3 and Bcl-2 were determined by western blotting. Cerebral infarct volume and motor function were evaluated by cresyl violet staining and foot fault test respectively. The results showed that early exercise decreased the number of apoptotic cells (118.74 ± 6.15 *vs.* 169.65 ± 8.47, *p* < 0.05, *n* = 5), inhibited the expression of caspase-3 and cleaved caspase-3 (*p* < 0.05, *n* = 5), and increased the expression of Bcl-2 (*p* < 0.05, *n* = 5). These data were consistent with reduced infarct volume and improved motor function. These results suggested that early exercise could provide neuroprotection through inhibiting neuron apoptosis.

## 1. Introduction

Cerebral ischemia (stroke) is one of the most serious neurological disorders. Although advanced technologies and facilities have significantly increased the survival rate of stroke patients in the past decades, most survivors suffer from permanent disability, including impairments in motor, communication, cognitive, learning and memory [1]. Thus stroke not only reduces daily quality of life, but also places a heavy burden on family and community [2]. Unfortunately, to date we still lack therapeutic strategies that can effectively improve clinical outcome [3]. Increasing evidence shows that exercise is an effective rehabilitation program for treatment of stroke [4]. Clinic practices suggested that exercise after stroke could improve the motor and sensory function and prevent the decline of cognitive ability [5]. However, the conventional exercise protocol was initiated at delayed phase after stroke, such as one week later in rats, and the validity of early exercise remained controversial [6]. Some studies showed that conditioned use of the affected limb and excessive intense exercise at early phase after stroke exacerbated ischemia injury [7,8]. However, some recent reports showed that moderate exercise at early stage after stroke protected against brain injury in experimental rats [9,10]. Furthermore, some clinical data showed that stroke patients with early exercise had a better functional outcome [11–13]. Importantly, early exercise has been recommended in the Clinical Guidelines for Stroke Management 2010 document sponsored by the National Stroke Foundation in Australia [14]. Thus these data indicated that the protocol of early exercise is critical to functional recovery, and moderate intensity early exercise was effective for functional recovery. However, the underlying mechanism responsible for neuroprotection of early exercise remains poorly understood.

The death and loss of neurons is the disastrous consequence induced by cerebral ischemia. Within a few minutes after ischemia, the neurons in ischemic core undergo irreversible injury. The cell death was traditionally thought to occur exclusively via necrosis; recent research indicated that apoptosis was the important cause of neuron death [15], especially the cells in penumbra [16,17]. Clinical evidence showed that apoptosis occurred in the penumbral region from 1 to 26 days after stroke [18,19]. Thus, suppressing apoptosis at early stage after stroke may be an important opportunity for salvage of neurons in penumbra, and then reduce the infarct volume and alleviate brain injury induced by ischemia [20–22].

Increasing evidence suggested that appropriate exercise could suppress apoptosis induced by ischemia [23], stress [24] and toxicant [25,26]. It is now well established that regular exercise can provide cardioprotection against ischemia-reperfusion and aging. Some recent research indicated that reduced apoptosis of cardiac muscles was one of the underlying mechanisms [27,28]. Exercise training decreased the generation of reactive oxygen species and the expression of pro-apoptotic proteins including Bax, active caspase-3/8/9, concurrently increased the expression of anti-apoptotic protein such as Bcl-2, HSP70/72 and SOD [29–32]. Additional, exercise training reduced the cardiac apoptosis in obese rats [33] and doxorubicin-induced rats [25,34]. In the field of central neural system, a few reports indicated that moderate exercise increased the concentration of HSP72 in plasma [35], reversed stress-induced Bax oligomer formation (a key pro-apoptotic complex) in the cerebral cortex [24], and attenuated the release of pro-inflammatory cytokines [36]. Using a transgenic Alzheimer’s disease (AD) mice model, Um *et al.* found that exercise inhibited the apoptotic cascades, including Bax, cytochrome c, caspase-9 and caspase-3, increased the expression of heat-shock protein-70 (HSP-70) and glucose-regulated protein-78 (GRP-78), and decreased amyloid beta-42 (Abeta-42) peptides significantly [37]. Our recent reports showed that early exercise increased mitochondrial biogenesis, reduced infarct volume and protected against brain injury [38,39].

Thus, it is reasonable to hypothesize that early exercise is able to decrease the number of apoptotic cells induced by focal cerebral ischemia/perfusion. In order to test this hypothesis, in the present study we examined the effect of early exercise on apoptosis in an experimental cerebral ischemic rat model.

## 2. Results and Discussion

### 2.1. Physiological Variables

Physiological variables were monitored before, during, and after stoke (middle cerebral artery occlusion, MCAO). There were no significant differences in pH (hydrogen ion concentration) values, paCO_2_ (partial pressure of carbon dioxide in artery blood), paO_2_ (partial pressure of oxygen in arterial blood), and MABP (mean arterial blood pressure), or rectal temperature among exercise with ischemia, non-exercise with ischemia and the sham groups before, during, and after MCAO operation (Table 1).

### 2.2. Early Exercise Reduced Apoptotic Cells after MCAO

**Figure 1:**
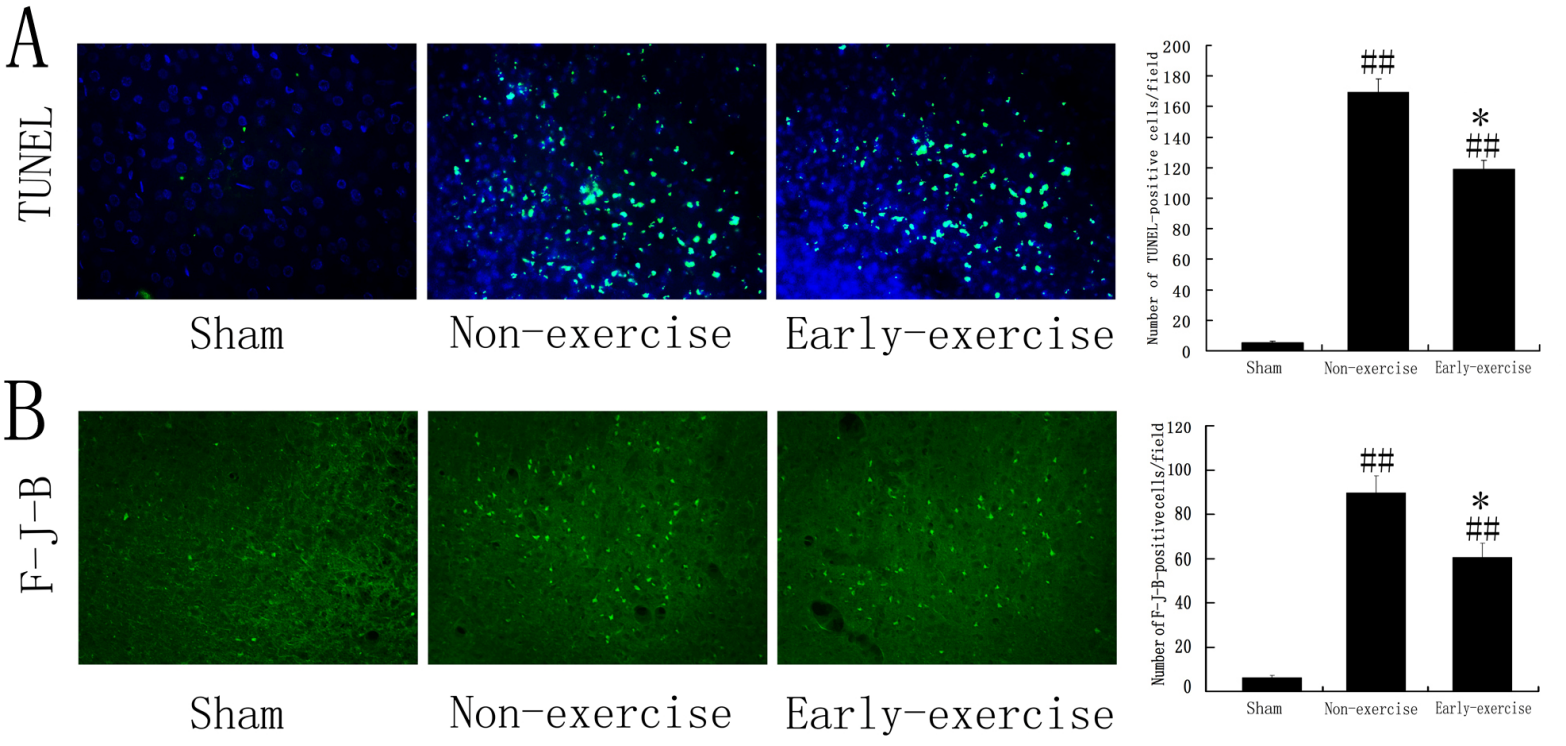
Early exercise significantly reduced apoptotic cells detected by TdT-mediated dUTP-biotin nick-end labeling (TUNEL) and Fluoro-Jade-B staining. (**A**) Representative photomicrographs of apoptotic cells detected by TUNEL staining in penumbra of cortex and quantitative histogram, the green signal represented TUNEL positive nucleus and the blue signal represented normal nucleus; (**B**) Representative photomicrographs of degenerating neurons detected by Fluoro-Jade-B staining in penumbra of cortex and quantitative histogram, the green signal represented Fluoro-Jade-B positive cells (degenerating neurons). *n* = 5 for each group. * reprsents *p* < 0.05 *vs.* the non-exercise group; # reprsents *p* < 0.05; ## reprsents *p* < 0.01 *vs.* sham group. ×200 magnification.

Ischemia induced a significant neural cells apoptosis in penumbra, as shown in Figure 1A. We detected the distribution and the number of apoptotic cells in penumbra of the cortex at 14th day after ischemia/reperfusion using TdT-mediated dUTP-biotin nick-end labeling (TUNEL) staining. Quantitative analysis showed that focal cerebral ischemia markedly induced cells apoptosis (Figure 1A, green signal represented TUNEL positive nucleus and blue signal represented normal nucleus). Early exercise significantly reduced the number of TUNEL positive cells in penumbra of cortex compared to non-exercise control group (118.74 ± 6.15 *vs.* 169.65 ± 8.47, *p* < 0.05, *n* = 5 for each) (Figure 1A). In contrast, we observed only a few scattered apoptotic cells in sham group.

Fluoro-Jade-B is an anionic fluorescent probe that can selectively stain degenerating neurons with high sensibility [40]. Here we further detected the degenerating neurons induced by MCAO through Fluoro-Jade-B staining. A similar trend was observed in the number of degenerating neurons as shown in Figure 1B. Compared to non-exercise control group, early exercise significantly decreased the Fluoro-Jade-B positive cells in penumbra (60.45 ± 6.58 *vs.* 89.73 ± 7.62, *p* < 0.05, *n* = 5 for each) (Figure 1B). Similarly, only a few apoptotic cells were detected in sham group.

### 2.3. Expression of Caspase-3, Cleaved Caspase-3 and Bcl-2

**Figure 2:**
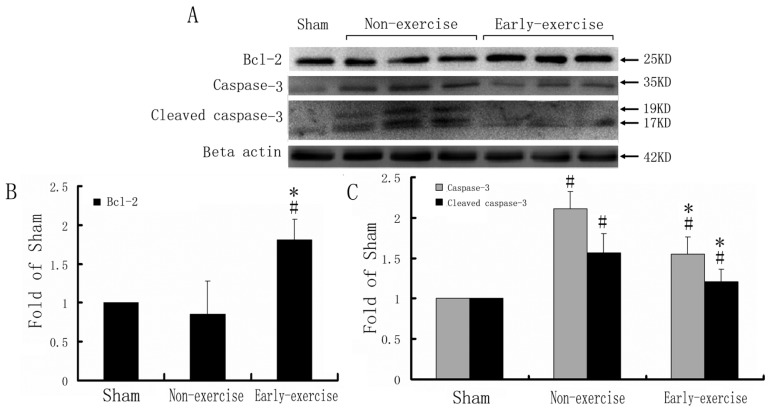
Expression of caspase-3, cleaved caspase-3 and Bcl-2. (**A**) Protein bands of caspase-3, cleaved caspase-3 and Bcl2 in cortex after MCAO detected by western blotting; (**B**) Semi-quantitative results for caspase-3, cleaved caspase-3 and Bcl2, respectively. *n* = 5; for each group. * *p* < 0.05, *vs.* the non-exercise group; # *p* < 0.05, *vs.* sham group.

Active caspase-3 is a key component of death-execution cascades in apoptotic progress. If the decreased apoptosis after ischemia resulted from caspase-3 signaling pathway, significant change should be observed. Thus we detected the expression of caspase-3 and cleaved caspase-3 (active caspase-3) by western blotting at day 14 after MCAO. While ischemia markedly up-regulated the expression of caspase-3 and cleaved caspase-3, early exercise significantly decreased the expression of caspase-3 and cleaved caspase-3 compared to non-exercise control group (*p* < 0.05) (Figure 2A,C), which was consistent with the results observed by immunohistochemistry (TUNEL and Fluoro-Jade, Figure 1A,B). In contrast, early exercise significantly increased the expression of Bcl-2, an important anti-apoptosis protein (*p* < 0.05) (Figure 2A,B).

### 2.4. Early Exercise Reduced the Infarct Volume after MCAO

**Figure 3:**
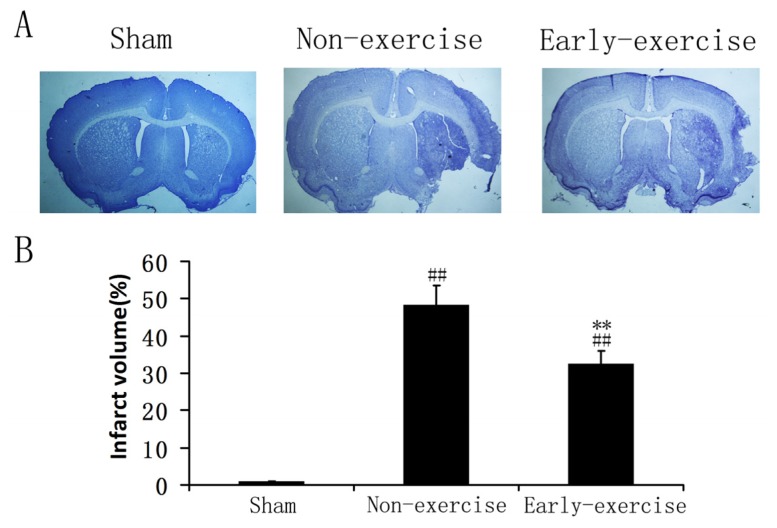
Early exercise reduced the infarct volume induced by focal ischemia. (**A**) Representative photomicrographs of cresyl violet (CV) staining; the cells in ischemic core exhibited dark cell bodies; (**B**) Quantitative results of infarct volume. *n* = 5 for each group. * *p* < 0.05, *vs.* the non-exercise group; ## *p* < 0.01, *vs.* sham group.

Focal ischemia induced a remarkable tissue loss because of necrotic and apoptotic cells in cortex and striatum (Figure 3A), and the suppressed apoptosis should result in a reduced infarct volume after stroke. Thus we measured the infarct volume at day 14 after MCAO. The results proved the hypothesis and showed that early exercise remarkably reduced the infarct volume compared to non-exercise group (32.27 ± 3.43% *vs.* 48.21 ± 5.24%, *p* < 0.05) (Figure 3A,B). In contrast, the rats in the sham group did not show any infarct area (Figure 3).

### 2.5. Early Exercise Improved the Recovery of Coordinated Locomotor Function after MCAO

**Figure 4:**
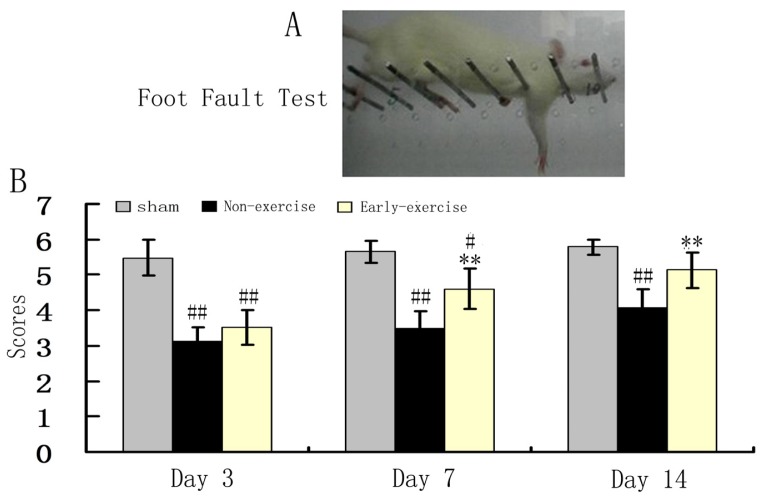
Early exercise improved the recovery of coordinated locomotor function. (**A**) A representative photograph of foot fault test, 0 score was given in this picture; (**B**) Quantitative results of foot fault test. *n* = 10 for each group. ** *p* < 0.01, *vs.* the non-exercise group; # *p* < 0.05; ## *p* < 0.01, *vs.* sham group.

The neurological defect of the contralateral forelimb to the lesion area is one of the major symptoms induced by MCAO. In order to evaluate the effect of early exercise on the functional recovery, coordinated locomotor function of fore limb was therefore determined by foot fault test at 3rd, 7th day and 14th day after MCAO. These results revealed that focal ischemia led to distinct right forelimb dysfunction at all test points (Figure 4). The performance of the exercise group and non-exercise group was no different at day 3 after MCAO and significantly lower than the sham group. The performance improved significantly in exercise group compared to those in the non-exercise group at 7th and 14th day (Figure 4B). Notably, rats in exercise group recovered coordinated locomotor function to the level of baseline at day 14, there was no difference compared to those in sham. In contrast, none of the rats in the sham control group exhibited neurological defects.

### 2.6. Discussion

To date, it is well known that apoptosis plays a key role in cell death after cerebral ischemia. Cerebral ischemia induced a rapid neural cell death in ischemic region, but the cell death in ischemic core is different from that in penumbra surrounding the ischemic core. Most cells in ischemic core undergo irreversibly necrosis, but many cells in ischemic penumbra will undergo apoptosis. Thus the cells in ischemic penumbra are potentially recoverable and there is an opportunity for salvage through some effective therapeutic methods.

Cerebral ischemia triggers apoptosis process through intrinsic and extrinsic pathways. Both result in the activation of caspase-3, which in turn cleaves an important DNA repair enzyme, poly ADP-ribose polymerase (PARP), and ultimately leads to DNA fragmentation and cell death [41]. Studies both in animal and human had demonstrated the up-regulated expression of caspase-3 and its effects after stroke [42,43]. Thus the activation of caspase-3 was crucial in the apoptotic cascade. A study using experimental cerebral ischemic animal model showed that deletion and inhibition of caspase-3 provided a significant neuroprotective effect through reduced cerebral infarct volume and prolonged the therapeutic window [44,45]. In the present study, we observed that early exercise remarkably decreased the expression of caspase-3 and cleaved caspase-3 at 14th day after experimental cerebral ischemia. This result indicated that early exercise protected against stroke through reduction of active caspase-3.

On the other hand, affected cells can initiate a pro-survival pathway to antagonize the apoptosis induced by stroke. Bcl-2 is an important anti-apoptotic protein which prevents the pore formation on outer mitochondrial membranes through binding with Bax, thereby inhibiting apoptosis derived from mitochondria pathway [46,47]. Chen and coworkers found that Bcl-2 expression in ischemia resistant neurons was higher than ischemia sensitive neurons [48]. However, the Bcl-2 expression of spontaneous recovery was insufficient to rescue the ischemic brain tissue and promote the functional outcome. Many studies have shown that Bcl-2 overexpression via gene transfer [49,50], bone marrow stromal cells administration [51] and ischemic postconditioning [52] suppressed the apoptosis and reduced the neuron loss in the ischemia penumbra. Consistent with these findings, our results indicated that ischemic rats with early exercise had a reduced apoptosis and reduced infarct volume, which were positively related to the higher Bcl-2 expression.

Mitochondrion is one of the most important organelles which provide energy to maintain the normal cell function. Cerebral ischemia and reperfusion damages the electrochemical gradient that is necessary for respiration and glucose oxidation in mitochondrial membrane [53,54]. Damaged mitochondrion not only generates the reactive oxygen species (ROS), but also releases cytochrome c and apoptotic induced factor (AIF) to initiate the apoptosis. Hence, strategies that prevent mitochondrial dysfunction and stimulate mitochondrial biogenesis should be considered as a potential neuroprotective way to reduce the apoptosis and promote functional recovery. Although there is currently no direct experimental evidence showing that exercise decreased the mitochondria dysfunction, increasing evidence indicates that exercise accelerates the mitochondrial biogenesis in skeletal muscle of normal animal [55,56]. A recent study demonstrated treadmill training up-regulated the expression of PGC-1 and citrate synthase and increased mitochondrial DNA in rodent brain [57,58]. Our recent results showed that early exercise promoted the mitochondrial biogenesis via increased expression of PGC-1, NRF-1 (mitochondrial-specific transcription factors) and COXIV (mitochondrial-specific protein) in an experimental stroke model [39]. These results were also positively correlated with reduced infarct volume and promoted functional outcome. These studies implied that improved mitochondrial function induced by early exercise may be the critical reason of inhibited apoptosis, but the detailed mechanism and direct experimental evidence needs to be further explored.

Neuroinflammatory response is another central event in ischemic brain injury. Increasing evidence has shown that neuroinflammatory response markedly exacerbated neurodegenerative cascades and damaged mitochondrial respiration [59,60]. Despite there being no direct evidence that inflammatory response is one of the inducers of apoptosis, ample evidence suggests that inflammation is closely related to apoptosis in multiple diseases. On the one hand, inflammatory response and apoptosis shared some common mechanisms. For example, proinflammatory cytokine (interleukin-1β and TNF-α) and caspase-1/7 participates in both inflammation and apoptosis [61–63]. On the other hand, although perfect apoptosis does not induce an inflammatory response, failed clearance of apoptotic cells promotes the release of proinflammatory cytokins and initiates inflammation [64]. Our recent study showed that early exercise reduced the infarct volume and improved the functional outcome through inhibiting the acute neuroinflammatory response [38]. These results were consistent with data in the present study.

Although our data indicated that early exercise suppressed the apoptosis and provided neuroprotection against ischemic brain injury, the exercise protocol used at early phase after stroke is very important to functional recovery. High intensity exercise or excessive use of the affected limb might exacerbate ischemia injury [7,8]. The moderate intensity and skilled training protocol was an effective early exercise for stroke treatment, such as Rota-rod training [9,38]. Therefore, the appropriate intensity and the combination of various exercise protocols at early phase after stroke might be a more scientific rehabilitative protocol in clinical practice.

There was a limitation in the present study. A sham group with exercise was absent in our experimental design which might be helpful to compare the effect of exercise on apoptosis and functional outcome between the normal and the ischemic condition. Despite this, our results indicated that early exercise significantly reduced the apoptosis induced by focal cerebral ischemia/reperfusion compared to ischemic control group.

## 3. Experimental Section

### 3.1. Rat Middle Cerebral Artery Occlusion (MCAO) Model

All animal experiments were approved by the animal experimental committee of Fudan University at Shanghai, China. Male Sprague-Dawley rats (250–270 g, Shanghai SLAC Laboratory Animal Co. Ltd., Shanghai, China) were housed under a 12:12 h light:dark cycle at 21 ± 1 °C, and food and water available *ad libitum*. Transient focal cerebral ischemia (tFCI) was induced by MCAO as previously described [38,65]. Briefly, rats were anesthetized with 1.5% isoflurane (Abbott, Abbott Park, IL, USA) and mechanically ventilated by an animal endotracheal tube. Then the left common carotid artery was separated and exposed, middle cerebral artery was occluded by a 4–0 nylon monofilament coated with a silicone tip, the monofilament was inserted into the internal carotid artery from the external carotid artery until mild resistance was felt. Reperfusion was established by completely withdrawing the nylon monofilament after 60 min of occlusion. Physiologic variables (pH, blood pressure, blood gases) were measured before, during, and after MCAO from left femoral artery. A thermostat-controlled heating blanket was used to maintain the rectal temperature at 37.0 °C during the whole operation. Rats in sham control group received all operation steps except for the occlusion of the middle cerebral artery.

### 3.2. Group and Treadmill Training Protocol

All rats were randomly divided to three groups, exercise with ischemia group, and non-exercise with ischemia group and sham group (without exercise and ischemia). Each group included 10 rats (5 used to detect apoptosis and infarct volume, and 5 used to western blotting, and all of rats received foot fault test). Treadmill training protocol was performed according to our recently published paper [38]. Briefly, all rats were habituated to the treadmill at 6–9 m/min for 3 consecutive days (10 min per day) before the operation. 24 h after reperfusion, rats in exercise with ischemia group received a 14 consecutive day’s exercise training on an electric treadmill (Litai Biotechnology Co. Ltd., Shandong, China, 30 min per day). The gradually increased intensity was set according to a previously described protocol [38]. Briefly, The intensity and duration was 5 m/min for the first 10 min, 9 m/min for 10 min, and 12 m/min for the last10 min at first day; The intensity and duration was 5 m/min for the first 5 min, 9 m/min for 5 min, and 12 m/min for last 20 min at second day; The intensity and duration was 12 m/min for 30 min from 3rd to 14th day. The slope was set at 0° for all phases of training. The rats in non-exercise with ischemia group and sham group were placed on the treadmills for the same duration, but were not made to run.

### 3.3. Tissue Section Preparation

At the end of exercise (14th day), five rats in each group were deeply anesthetized with 10% chloral hydrate (360 mg/kg, *i.p.*) and transcardially perfused with physiological saline followed by 4% paraformaldehyde. Then, brains were gained and transferred into a 20% sucrose solution for dehydration. Thereafter, frozen coronal brain sections were cut on a cryostat (30 μm in thickness). These slices were used for detection of the terminal deoxynucleotidyl transferase-mediated dUTP nickend labeling (TUNEL) staining, Fluoro- Jade B (F-J-B) staining and determination of infarct volume.

### 3.4. TUNEL and F-J-B Staining

TUNEL staining was used to detect cell apoptosis in ischemic penumbra according to the manufacturer’s protocol of FragEL™ DNA Fragmentation Detection Kit, Fluorescent-TdT Enzyme (Calbiochem, Darmstadt, Germany). In brief, four frozen coronal brain sections located in same region of each rat were incubated with proteinase K (20 μg/mL) for 10 min at room temperature followed by three washes in TBS, thereafter, these sections were incubated with TdT equilibration buffer for 30 min and then treated with the TUNEL reaction mixture in a dark humidor for 90 min at 37 °C. After three washes in TBS, The sections were covered with mounting media. Under an exciting light of 488 nm, these cells emitted yellow-green fluorescence in the nucleus were severed as apoptosis cells. The average number of apoptosis cells in five random visual fields per section (×200 magnification) in penumbra was used for statistical analysis.

In order to specifically detect the degenerate neurons in ischemic penumbra, F-J-B staining was performed as previously described [40]. Briefly, frozen brain sections were treated by 1% sodium hydroxide solution in 80% alcohol for 5 min. then these sections were immersed in 70% alcohol for 2 min and in distilled water for another 2 min followed by treatment in 0.06% potassium permanganate for 10 min. After 2 min wash in distilled water, they were then stained in 0.0004% Fluoro-Jade B (Histo-Chem Inc., Jefferson, AR, USA) staining solution for 20 min. then these sections were rinsed in distilled water followed by drying at 50 °C for 30 min. The dry sections were cleared in xylene and mounted by neutral balsam. Fluorescence image were obtained by a fluorescence microscopy (Nikon, Sendai, Japan) and the F-J-B positive cells appeared green fluorescence. The average number of F-J-B positive cells in five random visual fields per section (×200 magnification) in penumbra was used for statistical analysis.

### 3.5. Protein Isolation and Western Blotting

At the end of exercise (day 14), five rats in each group were sacrificed, the infarct cortex ipsilateral to the occluded side was isolated on ice. Then the cortex tissues were homogenized by RIPA lysis buffer (Beyotime Biotechnology, Shanghai, China) followed by 14,000*g* centrifugation at 4 °C for 20 min. Supernatants were separated by gel electrophoresis and transferred onto polyvinylidene fluoride (PVDF) membranes (Millipore, Boston, MA, USA). After 1 h block in 5% *w*/*v* bovine serum albumin (Roche, south san Francisco, CA, USA), the membrane was incubated in primary antibody against Bcl-2 (Cell Signaling Technology, Danvers, MA, USA; 1:2000), caspase-3 and cleaved caspase-3 (Cell Signaling Technology, Danvers, MA, USA; 1:500 and 1:200 respectively), beta actin (Sigma, St. Louis, MS, USA; 1:2000). 24 h later, the membrances were washed and incubated in horseradish peroxidase (HRP)-conjugated antirabbit IgG (Jackson, MS, USA, 1:2000) for 1 h. Thereafter, protein band was visualized by pierce ECL kit (Thermo Scientific, Pittsburgh, PA, USA) and semi-quantified by fluorescence densitometry with the BIO-RAD system (Bio-Rad, Hercules, CA, USA). The protein signals were normalized against the fluorescence densitometry of beta actin.

### 3.6. Determination of Brain Infarct Volume

Cresyl violet (CV) staining was used to determinate brain infarct volume as previously described [66]. In brief, frozen coronal brain sections (total 10 slices with a 360-μm interval for each rat through the sensorimotor cortex and striatum) were stained in 0.1% cresyl violet solution for 10 min at room temperature followed by quick differentiation in 1% glacial acetic acid (in 70% ethanol). These sections were then dehydrated and clarified through ethanol and xylene respectively, and mounted with neutral balsam. The infarct area was defined as the region with unstained area or containing dark pyknotic/necrotic cell bodies according to published paper [67,68]. The infarct area was traced and calculated with NIH Image software [69]. In order to minimize the error resulted by edema, the infract volume was determined by an indirect method [68]:

$$\begin{array}{c} \text{Infarct volume percent}=(\text{contralateral hemisphere area}-\text{normal region area in the}\\ \text{ipsilateral hemisphere})/\text{contralateral hemisphere area}\times 100\%. \end{array}$$

### 3.7. Foot Fault Test

At 3rd, 7th and 14th day after MCAO, coordinated locomotor function of affected forelimb (right forelimb) was quantitatively evaluated by foot fault test as previously described [38]. All rats were evaluated by a laboratory assistant blinded to experiment design according to a published criterion [45]. In brief, 6, correct placement; 5, partial placement; 4, correction, 3, replacement; 2, slight slip; 1, deep slip; 0, total miss. A higher score represented a better forelimb placement. For each rat, the average scores of three trials were used for statistical analysis.

### 3.8. Statistical Analysis

All data were presented as mean ± standard error of the mean (SEM). Statistical differences were assessed using one-way analysis of variance (ANOVA) followed by post hoc Fisher’s PLSD tests. *p* < 0.05 was considered statistically significant.

## 4. Conclusions

Early exercise can provide neuroprotection through reduced infarct volume and improved motor coordination. The underlying mechanism might be the reduced apoptosis in focal cerebral ischemia through inhibited caspase-3 and up-regulated Bcl-2. It is necessary to further elucidate the detailed molecular mechanism for the effective clinical application of early exercise.

## Figures and Tables

**Table 1:** Physiological variables among different groups before, during, and after middle cerebral artery occlusion (MCAO).

Group	pH	PaCO_2_ (mm Hg)	PaO_2_ (mm Hg)	MABP (mm Hg)	Rectal temperature (°C)
sham (*n* = 6)	7.36 ± 0.02	44.3 ± 1.8	95.8 ± 2.8	96.1 ± 6.7	37.3 ± 0.1
exercise (*n* = 10)					
pre-ischemia	7.30 ± 0.07	40.5 ± 6.7	89.9 ± 2.8	95.0 ± 4.5	37.6 ± 0.3
during-ischemia	7.32 ± 0.05	42.7 ± 8.1	84.3 ± 5.3	84.4 ± 9.4	37.5 ± 0.2
post-ischemia	7.30 ± 0.09	38.2 ± 8.4	89.5 ± 6.7	89.2 ± 10.0	37.6 ± 0.3
non-exercise (*n* = 10)					
pre-ischemia	7.39 ± 0.01	37.95 ± 2.48	87.65 ± 2.97	95.87 ± 3.60	37.5 ± 0.4
during-ischemia	7.36 ± 0.01	47.63 ± 8.24	82.86 ± 2.89	88.5 ± 2.78	37.3 ± 0.3
post-ischemia	7.4 ± 0.006	34.43 ± 1.8	88.4 ± 3.98	90.4 ± 3.5	37.6 ± 0.2

Data represent means ± standard error of the mean (S.E.M.).
